# When the model defines the message: methodological considerations in experimental lung warm ischemia

**DOI:** 10.3389/ti.2026.17803

**Published:** 2026-10-16

**Authors:** Jitte Jennekens, Daan Imholz, Sue Braithwaite, Linda de Heer, Arne Neyrinck

**Affiliations:** 1 Department of Cardiothoracic Surgery, University Medical Center Utrecht, Utrecht, Netherlands; 2 Department of Anesthesiology, University Medical Center Utrecht, Utrecht, Netherlands; 3 Anesthesia Research Unit, Department of Cardiovascular Sciences, KU Leuven, Leuven, Belgium

**Keywords:** experimental model, ischemia reperfusion injury, lung, transplantation, warm ischemia

We read the article by Premachandra et al., “Resilience of lung grafts to warm ischemia: Assessment by *ex vivo* perfusion using functional and molecular analysis,” with great interest [1]. We thank the authors for their comprehensive investigation of the effects of 2-h prolonged warm ischemia (WI) on lung graft function and biological responses during *ex vivo* lung perfusion (EVLP). The authors reported lower initial compliance and increased pulmonary vascular resistance in the WI group compared to control lungs. These differences resolved over the duration of the EVLP. At the end of EVLP, no significant differences in molecular and histological markers of injury were observed, except for concentrations of lactate and PECAM, which were higher in the control lungs.

Premachandra et al. positioned their study within the broader aim of establishing the tolerance limit of lungs to WI in a clinically relevant pig model. While some limitations have been appropriately addressed by the authors, other aspects warrant further consideration and discussion, especially the clinical relevance of their model. A relevant donation after circulatory death (DCD) model should address the critical aspects of the procedure and timeline because these factors also influence the effect of WI on the lungs. In clinical practice, extended periods of WI either occur during a prolonged agonal phase or in uncontrolled DCD settings, where the lungs are mechanically ventilated after failed resuscitation attempts until the “no touch” period [2, 3]. This is different from the WI model used by Premachandra et al., in which the lungs were left inflated in the cadaver for a 2-h period. It has been previously demonstrated that the mode of death in DCD donors affects lung graft quality, with hypoxic cardiac arrest associated with inferior graft quality compared to immediate circulatory arrest or exsanguination [4]. In the case of this study, the animals were euthanized by aortic clamping and simultaneous exsanguination. In addition, previous studies have shown that ventilated or inflated lungs are protected from WI injury, while deflated lungs developed pulmonary edema and impaired gas exchange during subsequent normothermic reperfusion [5–7]. In line with these findings, the authors mentioned that several experimental studies reported an enhanced inflammatory response with extended periods of WI (1–2 h) during EVLP. Premachandra et al. used methylprednisolone during EVLP and attributed the discrepant inflammatory findings primarily to the absence of methylprednisolone in the other studies. Although it has indeed been demonstrated that addition of methylprednisolone can reduce inflammatory effects, another important difference is the WI model [8]. The studies that the authors cited used deflated WI, a model associated with more extensive lung injury than the inflated WI model used by Premachandra et al. Therefore, the absence of inflammatory differences in their study may reflect not only the use of methylprednisolone but also the specific experimental model that has been used.

Finally, we would like to address the split-lung EVLP model. We were surprised that only left lungs were allocated to the WI group whereas right lungs were allocated to the control group. Consequently, treatment assignment is confounded by anatomical laterality, lung size, and perfusion and ventilation. Following the Toronto protocol, the authors adjusted perfusion and ventilation to 24% and 16% of the theoretical cardiac output and to tidal volumes of 3.5 and 2.5 mL/kg for the right and left lungs, respectively. This corresponds to a right: left perfusion distribution of 60%/40%. In humans, a perfusion distribution of 55%:45% is commonly assumed [9]. Accounting for size differences between the right and left lungs by adjusting flow and ventilation does not necessarily mean that the resulting *ex vivo* perfusion of right and left lungs is physiologically comparable, with the potential risk of being more protective in one group compared to the other. A similar principle may apply to perfusate-derived biomarkers. Interestingly, lactate and PECAM concentrations were higher in the control lungs at the end of EVLP. The authors proposed that this may reflect a protective priming effect of WI. We propose an alternative explanation: the greater lung mass and potentially greater amount of perfused tissue in the right lungs of the control group may have contributed to the differences in absolute marker release. Without normalization of perfusate markers to lung mass, or another measure of perfused tissue amount, differences in marker concentrations should be interpreted with caution. Future split-EVLP studies could randomize treatment assignment to the right and left lungs across animals [10, 11]. Taken together, this study demonstrated relatively limited functional and biological effects in porcine lungs exposed to 2-h inflated WI under a steroid-containing EVLP protocol. However, the specific experimental conditions used in EVLP studies should be carefully considered when assessing the clinical relevance of the observed findings.

## Data Availability

The original contributions presented in the study are included in the article/supplementary material, further inquiries can be directed to the corresponding author.
